# Case Report: Tail-of-the-curve advantage from immune checkpoint inhibitor–anti-VEGF combination therapy: extended survival in a patient with metastatic lung adenocarcinoma

**DOI:** 10.3389/fmed.2025.1714486

**Published:** 2025-12-12

**Authors:** Junxia Hu, Li Li, Xueyuan Mao, Xin Chang, Ying Liu, Lei Cao

**Affiliations:** 1 Department of Oncology, Jiangsu Province (Suqian) Hospital, Suqian, Jiangsu, China; 2 Department of Pathology, Jiangsu Province (Suqian) Hospital, Suqian, Jiangsu, China; 3 Department of Infection Control, Jiangsu Province (Suqian) Hospital, Suqian, Jiangsu, China; 4 Department of Oncology, Suqian Clinical Medical College of Jiangsu University, Suqian, Jiangsu, China

**Keywords:** lung cancer, chemotherapy, radiotherapy, immunotherapy, anti-angiogenic, tail-of-the-curve

## Abstract

Within the evolving landscape of cancer immunotherapy, the so-called tail-of-the-curve effect has emerged as a distinctive and clinically relevant phenomenon, defined by the persistence of disease remission long after discontinuation of therapy and thereby reflecting the durability of antitumour immune responses. Immunotherapy has become an indispensable component of systemic treatment for advanced non-small-cell lung cancer (NSCLC), with immune checkpoint inhibitor (ICI)-based combinations—particularly those incorporating anti-angiogenic agents—demonstrating not only robust but also durable clinical benefit across multiple settings. Against this backdrop, we describe the clinical course of a patient with invasive adenocarcinoma of the left upper lobe who underwent surgical resection followed by adjuvant pemetrexed-cisplatin chemotherapy. Two years later, an isolated perihilar recurrence was treated with radical radiotherapy in combination with recombinant human endostatin (Endostar) as a radiosensitizer, after which the patient received four further cycles of the original chemotherapy regimen and subsequently transitioned to pemetrexed maintenance. Thirteen months into maintenance therapy, brain metastases were detected; at this juncture, the patient received whole-brain radiotherapy together with combined sintilimab and Endostar therapy. Although systemic treatment was discontinued after 18 months, the patient maintained disease control for an additional 36 months, consistent with a pronounced tail-of-the-curve effect. This case raises the possibility of integrating ICIs, anti-angiogenic therapy, and focal radiotherapy to elicit durable survival benefit in patients with chemotherapy-refractory, unresectable advanced NSCLC, and further highlights the clinical significance of the immunotherapy-associated tail effect.

Lung cancer remains one of the most burdensome malignancies worldwide and is associated with a dismal prognosis (1). Global population-based survival analyses across 185 countries have demonstrated that lung cancer continues to rank first in both incidence and cancer-related mortality among men (2). Non-small-cell lung cancer (NSCLC) constitutes the predominant histological subtype, for which surgical resection remains the treatment of choice in early-stage disease when no contraindications are present. However, a considerable proportion of patients inevitably relapse despite complete resection followed by standard adjuvant chemotherapy, thereby highlighting the critical importance of developing rational and precise adjuvant as well as subsequent-line therapeutic strategies to further improve survival outcomes.

The patient was initially diagnosed with invasive adenocarcinoma of the left upper lobe (pT_2a_N_0_M_0_, stage IB), characterized by micropapillary components and visceral pleural invasion. Following the curative resection, adjuvant chemotherapy with four cycles of pemetrexed plus cisplatin was administered. Platinum-based doublet chemotherapy remains the most widely adopted adjuvant strategy for resected NSCLC, with recommended partners including paclitaxel and pemetrexed (the latter restricted to non-squamous histology) (3). Despite this standard treatment, the patient developed an isolated recurrence in the left hilar region after a two-year disease-free interval, consistent with oligometastatic relapse, and was subsequently managed with systemic chemotherapy combined with local radical radiotherapy and anti-VEGF radiosensitization. Endostar (recombinant human endostatin), an anti-angiogenic agent approved for advanced NSCLC, exerts its antitumor activity through pleiotropic mechanisms. In addition to targeting canonical pathways such as VEGF/VEGFR, FGF/FGFR, and PDGF/PDGFR, Endostar also modulates hypoxia-inducible factor-1α, matrix metalloproteinases, and integrin αvβ3. By suppressing endothelial cell migration and inhibiting neovascularization, it deprives tumor cells of essential nutrients, thereby limiting both proliferation and metastatic spread (4). The patient subsequently received maintenance therapy with pemetrexed monotherapy, which was continued for 13 cycles until an isolated brain metastasis was identified during follow-up. At this juncture, radical local radiotherapy was administered to the brain lesion, concurrently with combination therapy comprising sintilimab and Endostar. Immune checkpoint inhibitors (ICIs) have recently transformed the therapeutic landscape of advanced NSCLC, with agents such as sintilimab demonstrating meaningful efficacy by blocking the PD-1/PD-L1 axis, thereby reversing tumor-induced immune evasion and restoring T-cell–mediated antitumour immunity (5). The concurrent use of anti-VEGF therapy can further remodel the tumor microenvironment by normalizing blood vessels, reducing infiltration of immune suppressor cells (e.g., Tregs, MDSCs), and enhance the sensitivity of tumor cells to immunotherapy, thus providing a strong biological rationale for this combined approach.

The present case highlights a patient with advanced NSCLC who, after relapse following standard chemotherapy, experienced a pronounced and durable tail-of-the-curve benefit with immune checkpoint inhibitor–anti-angiogenic combination therapy. These findings not only underscore the potential of such regimens to achieve long-term disease control in selected patients but also offer new insights into the optimization of comprehensive treatment strategies for advanced NSCLC.

## Case presentation

**Figure 1:**
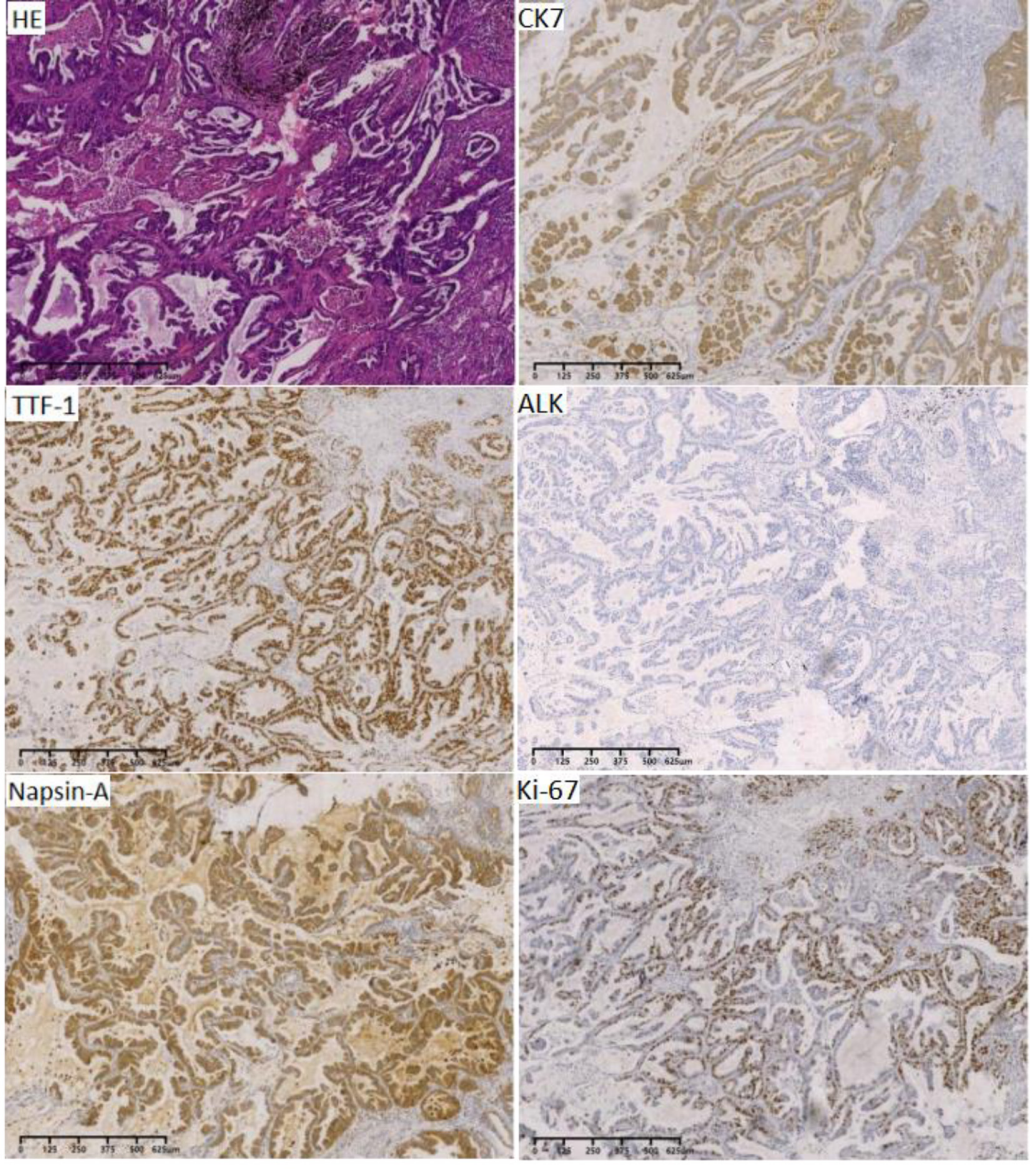
Pathological stained section (magnification: *40). HE staining consistent with invasive adenocarcinoma; TTF-1 (+++); Napsin-A (+++); CK7 (++); ALK (–); KI-67 (40% +).

**Figure 2:**
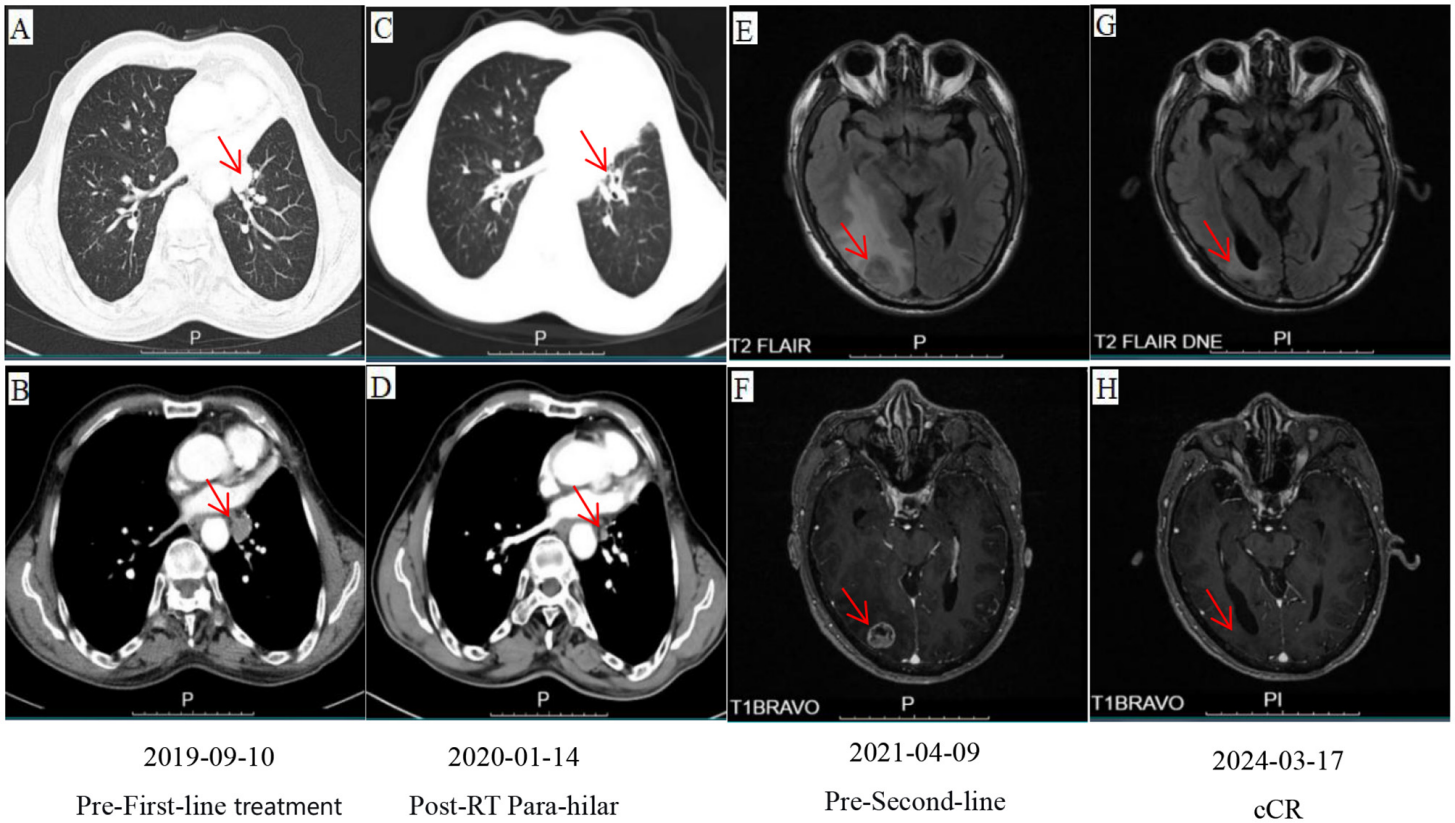
**(A, B)** CT images of the lung window **(A)** and the mediastinal window **(B)** on September 10, 2019; **(C, D)** CT images of the lung window **(C)** and the mediastinal window **(D)** on January 14, 2020; **(E, F)** MRI images of T2 FLAIR **(E)** and T1 BRAVO **(F)** on April 9, 2021; **(G, H)** MRI images of T2 FLAIR **(G)** and T1 BRAVO **(H)** on March 17, 2024.

**Figure 3:**
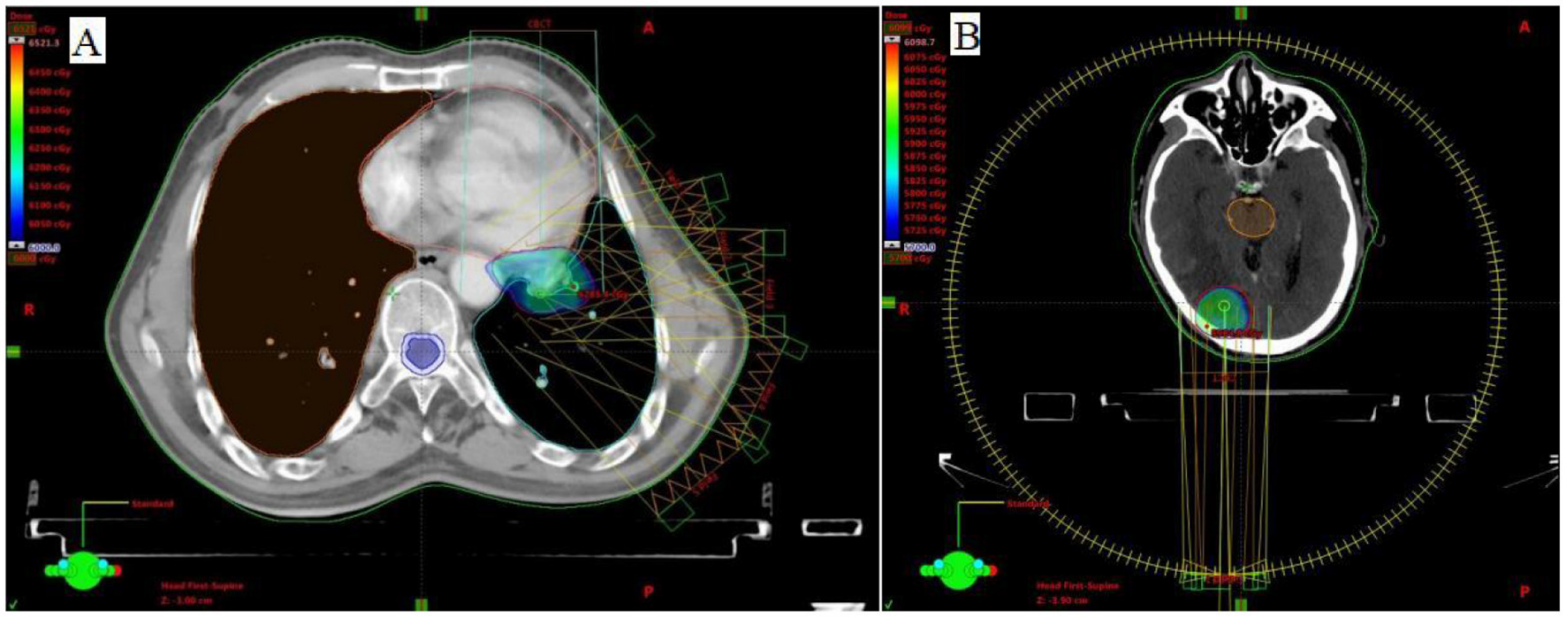
**(A)** Radiotherapy Planning Diagram for Para-hilar Metastasis from 2019-11-06 to 2019-12-17; **(B)** Radiotherapy Planning Diagram for Brain Metastasis from 2021-04-13 to 2021-05-07.

**Figure 4:**
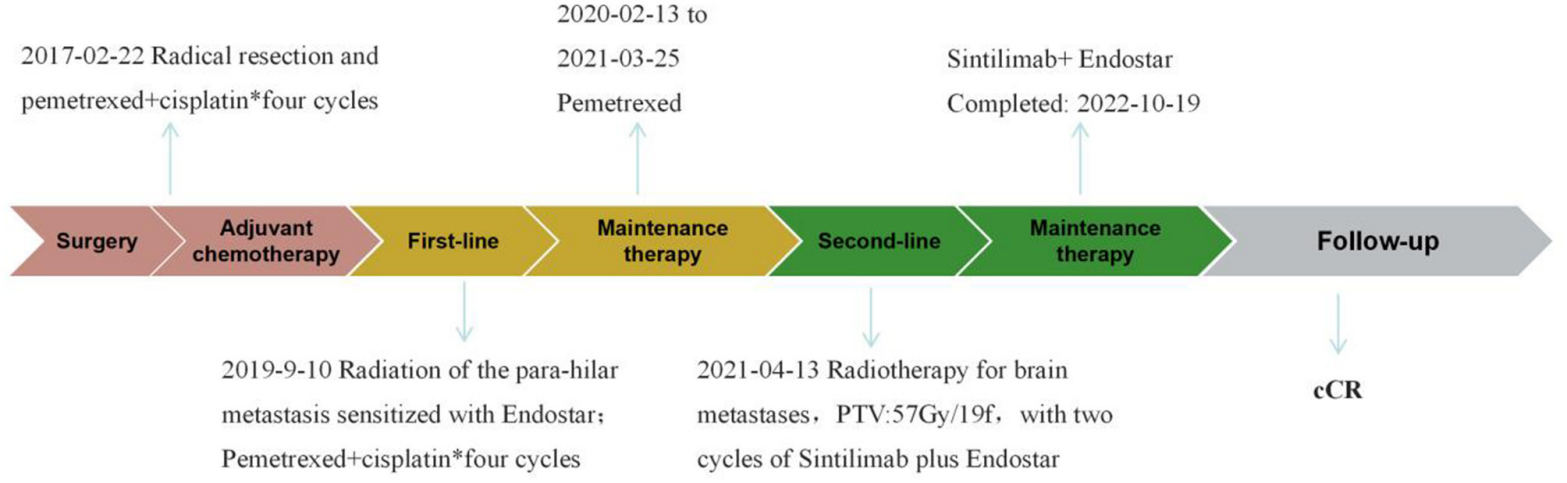
Patient treatment timeline.

A 67-year-old male initially presented with left upper lobe lung cancer and underwent radical lobectomy on February 22, 2017. Histopathological examination confirmed invasive adenocarcinoma (grade I) with both papillary and micropapillary components. The tumor measured 2.5 × 2.5 × 2.0 cm, demonstrated visceral pleural invasion without penetration, and the bronchial margin was negative. No lymph node metastases were detected in stations 10 and 11 (0/1, 0/3, 0/1). Immunohistochemical profiling (Figure 1) supported the diagnosis of invasive adenocarcinoma, with tumor cells positive for Ki-67 (40%), TTF-1 (+++), CK19 (+++), CK8/18 (+++), CK7 (++), Napsin-A (+++), and EGFR (+++), while negative for ALK. The patient subsequently received four cycles of adjuvant chemotherapy with pemetrexed plus cisplatin beginning on March 22, 2017. Surveillance imaging on March 25, 2019, revealed a left lower lobe nodule adjacent to the hilum, measuring 15 × 14 mm. Follow-up CT on September 10, 2019 (Figures 2A, B), demonstrated interval growth to 26 × 16 mm, consistent with disease recurrence. Molecular testing of an 11-gene lung cancer panel, including EGFR, ALK, KRAS, ROS1, MET, ERBB2, BRAF, RET, NTRK1, NTRK2, and NTRK3, yielded negative results. Given evidence of localized progression, systemic chemotherapy with pemetrexed (0.8 g d_1_) and cisplatin (60 mg d_1 − 2_, q3w) was reinitiated and administered for four cycles between September 2019 and January 2020. Concurrently, from November to December 2019, radical radiotherapy was delivered to the left hilar lesion (DT: 60 Gy in 30 fractions, PTV encompassing the recurrent mass; Figure 3A), during which Endostar was administered as a radiosensitizer. Reassessment with CT on January 14, 2020 (Figures 2C, D), demonstrated a partial response (PR) per RECIST. The patient then transitioned to pemetrexed maintenance therapy (0.8 g d_1_, q3w), which was continued for 13 cycles from February 2020 to March 2021. In April 2021, the patient developed a headache and progressive memory decline. Brain MRI on April 9, 2021 (Figures 2E, F), revealed a right occipital lobe lesion measuring 21 × 18 mm with extensive perilesional edema, consistent with intracranial metastasis. Meanwhile, CT indicates the disappearance of left hilar lesion. From April 13 to May 7, 2021, radical radiotherapy was administered to the brain lesion (DT: 57 Gy in 19 fractions of 3 Gy each; Figure 3B). During radiotherapy, the patient concurrently received two cycles of sintilimab (200 mg d_1_) combined with Endostar (30 mg, continuous intravenous infusion over 24 hours for 7 days). Upon completion of radiotherapy, maintenance therapy with sintilimab (200 mg) and Endostar (30 mg CIV 24 h for 7 days, q3w) was continued until October 19, 2022. Follow-up imaging (Figures 2G, H) demonstrated a complete clinical remission (cCR). The patient discontinued treatment thereafter and has remained under regular surveillance. At the time of this report, he continues to maintain durable progression-free survival, highlighting a clinical course consistent with the long tail-of-the-curve effect (Figure 4).

## Discussion

In this case, the patient experienced disease progression after 13 months of first-line maintenance therapy, necessitating a prompt transition in systemic management. Given the emergence of localized brain metastasis, intensity-modulated radiotherapy (IMRT) for the limited brain metastases, and no prophylactic radiotherapy was performed for other parts of the cranium. In the absence of actionable driver mutations, systemic therapy was switched to a regimen combining immune checkpoint blockade with anti-angiogenic therapy (sintilimab plus Endostar), initiated concomitantly with radiotherapy and subsequently continued as maintenance for 18 months. Remarkably, after treatment discontinuation, the patient sustained 36 months of recurrence-free survival—a trajectory consistent with a durable tail-of-the-curve effect that is likely driven primarily by the long-tail benefit of immunotherapy and the potentiating effect of anti-VEGF therapy.

The tail effect represents a defining feature of immunotherapy, distinguished by the persistence of clinical benefit long after cessation of treatment and reflective of durable antitumour immune memory (6). Mechanistically, this phenomenon is underpinned by the activity of specialized memory T-cell subsets within the tumor microenvironment. Tissue-resident memory T cells (TRM) are maintained through continuous expression of tissue-retention molecules such as CD103 and CD49a, supported by cytokine signals including TGF-β and IL-15 (7, 8). Upon antigen recognition, TRM cells rapidly reactivate through TCR–pMHC signaling, triggering MAPK/ERK and NF-κB pathways and releasing effector molecules such as IFN-γ and granzyme B, thereby exerting direct cytotoxicity against tumor cells. In parallel, stem-like memory T cells (TSCM) and central memory T cells (TCM) preserve self-renewal and longevity via Wnt/β-catenin and PI3K/Akt/mTOR signaling, while IL-7/STAT5 maintains survival (7, 9, 10). Upon antigen rechallenge, these subsets expand into effector T cells, replenishing the memory pool and sustaining long-term immune surveillance. At the epigenetic level, memory T cells retain chromatin “memory domains” marked by histone modifications, which preserve transcriptional accessibility for rapid reactivation of effector programs upon secondary antigen exposure (11–14). Moreover, humoral immunity may play a role in anti - tumor defense by performing multiple functions, including antigen processing and presentation, cytokine - mediated signaling, antibody class switching, expression, and secretion (15). Together, these mechanisms enable long-lived immune memory that clinically manifests as the tail effect. The clinical significance of this phenomenon lies in its ability to reshape the natural history of advanced malignancies. Whereas conventional radiotherapy and chemotherapy achieve tumor regression predominantly through direct cytotoxicity, often with limited durability, immunotherapy mobilizes endogenous immune responses capable of long-term maintenance, thereby conferring prolonged disease control and survival benefit (16, 17). Blockade of the PD-1/PD-L1 axis has emerged as a cornerstone of this strategy, reversing tumor-induced immune suppression by restoring T-cell proliferation and cytotoxic function (18–21). Although studies have shown that prior anti-VEGF therapy is a negative predictor of immune therapy response, we administered a combination of both therapies as maintenance after intracranial lesion radiotherapy (22). Beyond monotherapy, combinatorial regimens integrating immune checkpoint inhibitors with anti-angiogenic agents have demonstrated pronounced synergism (23, 24). In the present case, concurrent administration of sintilimab and Endostar with radiotherapy yielded durable disease control and long-term survival, consistent with the biological rationale underpinning this strategy.

Anti-VEGF therapy exerts its immunomodulatory effects by normalizing aberrant tumor vasculature, enhancing T-cell infiltration and activity, reducing the suppressive influence of regulatory T cells (Tregs) and myeloid-derived suppressor cells (MDSCs), and modulating cytokine networks to reprogram the tumor microenvironment toward an immunostimulatory state (25, 26). Meanwhile, checkpoint inhibition relieves PD-1–mediated suppression of T-cell activity, reactivating tumor-specific immune responses (5). The convergence of these mechanisms produces a synergistic antitumor effect: vascular normalization facilitates immune effector cell entry and function, while checkpoint blockade sustains their activity. Such synergy may extend therapeutic benefit even to patients with low PD-L1 expression (27), resulting in significant improvements in progression-free and overall survival.

This case illustrates the possibility of integrating radiotherapy, immune checkpoint inhibition, and anti-angiogenic therapy to achieve durable survival benefit in advanced NSCLC. The observation of a prolonged tail-of-the-curve effect highlights not only the capacity of immunotherapy to induce lasting immune memory but also the value of rational combinatorial strategies in reshaping outcomes for patients with chemotherapy-refractory disease.

## Data Availability

The original contributions presented in the study are included in the article/supplementary material, further inquiries can be directed to the corresponding author.
